# Conflicts at work are associated with a higher risk of cardiovascular disease

**DOI:** 10.3205/000249

**Published:** 2017-04-26

**Authors:** Louis Jacob, Karel Kostev

**Affiliations:** 1 Faculty of Medicine, University of Paris 5, Paris, France; 2 Epidemiology, IMS Health, Frankfurt, Germany

**Keywords:** workplace conflicts, cardiovascular diseases, myocardial infarction, general practices, Germany

## Abstract

**Background:** Only few authors have analyzed the impact of workplace conflicts and the resulting stress on the risk of developing cardiovascular disorders. The goal of this study was to analyze the association between workplace conflicts and cardiovascular disorders in patients treated by German general practitioners.

**Methods:** Patients with an initial documentation of a workplace conflict experience between 2005 and 2014 were identified in 699 general practitioner practices (index date). We included only those who were between the ages of 18 and 65 years, had a follow-up time of at least 180 days after the index date, and had not been diagnosed with angina pectoris, myocardial infarction, coronary heart diseases, or stroke prior to the documentation of the workplace mobbing. In total, the study population consisted of 7,374 patients who experienced conflicts and 7,374 controls for analysis. The main outcome measure was the incidence of angina pectoris, myocardial infarction, and stroke correlated with workplace conflict experiences.

**Results:** After a maximum of five years of follow-up, 2.9% of individuals who experienced workplace conflict were affected by cardiovascular diseases, while only 1.4% were affected in the control group (p-value <0.001). Workplace conflict was associated with a 1.63-fold increase in the risk of developing cardiovascular diseases. Finally, the impact of workplace conflict was higher for myocardial infarction (OR=2.03) than for angina pectoris (OR=1.79) and stroke (OR=1.56).

**Conclusions:** Overall, we found a significant association between workplace conflicts and cardiovascular disorders.

## Zusammenfassung

**Hintergrund:** Der Einfluss der Konflikte am Arbeitsplatz und des daraus resultierenden Stresses auf das kardiovaskuläre Risiko wurde bis jetzt nur selten untersucht. Das Ziel dieser Studie bestand in der Analyse der Assoziation zwischen Arbeitsplatzkonflikterfahrungen und kardiovaskulären Erkrankungen bei Patienten in hausärztlichen Praxen.

**Methoden:** Patienten mit initialer Dokumentation einer Arbeitsplatzkonflikterfahrung zwischen 2005 und 2014 (Indexdatum) wurden in 699 hausärztlichen Praxen identifiziert. Nur Patienten im Alter zwischen 18 und 65 Jahren, die über mindestens 180 Tage nach dem Indexdatum beobachtet werden konnten und keine Diagnosen von Angina Pectoris, Myokardinfarkt, koronarer Herzerkrankung und Schlaganfall vor dem Indexdatum hatten, wurden in die Analyse eingeschlossen. Insgesamt wurden 7.374 Patienten mit einer Arbeitsplatzkonflikterfahrung sowie 7.374 Patienten ohne Arbeitsplatzkonflikterfahrung (Kontrollgruppe) analysiert. Der Zielparameter war die Inzidenz von Angina Pectoris, Myokardinfarkt, koronarer Herzerkrankung und Schlaganfall abhängig von Arbeitsplatzkonflikterfahrungen.

**Ergebnisse:** Innerhalb der maximal fünf Beobachtungsjahre wurde bei 2,9% der Personen mit der Erfahrung eines Arbeitsplatzkonfliktes, aber nur bei 1,4% in der Kontrollgruppe eine kardiovaskuläre Erkrankung diagnostiziert (p-Wert <0,001). Arbeitsplatzkonflikterfahrung war mit 1,63-fach erhöhtem Risiko für eine kardiovaskuläre Erkrankung assoziiert Der Einfluss war viel höher für Myokardinfarktrisiko (OR=2,03) verglichen mit dem Risiko für Angina Pectoris (OR=1,79) und Schlaganfall (OR=1,56).

**Schlussfolgerungen:** Wir fanden eine signifikante Assoziation zwischen Arbeitsplatzkonflikterfahrungen und kardiovaskulären Erkrankungen.

## Introduction

Workplace mobbing is defined as “a situation in which one or several individuals persistently, and over a period of time, perceive themselves as being on the receiving end of negative actions from superiors or coworkers, and where the target of the bullying finds it difficult to defend him or herself against these actions” [1], [2], [3]. Despite some geographical preferences, mobbing, bullying, and harassment are interchangeable terms [4]. Workplace mobbing is known to have a negative impact on health [5] and mobbing at work has been significantly associated with depression, anxiety, and sleep disorders [6], [7], [8].

Only few authors have analyzed the impact of workplace mobbing and the resulting stress on the risk of developing cardiovascular disorders. A 2003 Finnish study found that victims of prolonged workplace mobbing were at a higher risk of being diagnosed with depression and cardiovascular diseases than the control subjects [9]. Later, it was discovered in a study of 738 Lithuanian teachers that regular mobbing at work was estimated to occur up to around 2.6% and was associated with cardiovascular diseases in the non-adjusted regression analysis [10]. More recently, an Australian study found that past and current exposure to negative workplace behavior, including mobbing, were significantly correlated with depression and negative cardiovascular outcomes [11].

Although the findings of these three studies are important, little is known about the impact of workplace conflicts on cardiovascular diseases. Furthermore, no data is yet available on the types of cardiovascular conditions that are more particularly affected by conflicts at work. Therefore, the goal of the present study was to analyze the association between workplace conflicts and cardiovascular disorders in patients treated by German general practitioners. 

## Methods

### Database

This study is based on data from the Disease Analyzer database (IMS Health), which compiles drug prescriptions, diagnoses, and basic medical and demographic data obtained directly and in anonymous format from computer systems used in physicians’ practices [12]. Diagnoses (ICD-10), prescriptions (Anatomical Therapeutic Chemical (ATC) Classification System), and the quality of reported data have been monitored by IMS based on a number of criteria (e.g., completeness of documentation and linkage between diagnoses and prescriptions). 

In Germany, the sampling methods used for the selection of physicians’ practices were consistent with a representative database of physician practices [12]. Prescription statistics for several drugs were very similar to data available from pharmaceutical prescription reports [12]. The age groups for given diagnoses in the Disease Analyzer were also commensurate with those in corresponding disease registries [12].

Finally, the Disease Analyzer database has already been effectively used in studies focusing on workplace mobbing [7] or cardiovascular disorders [13], [14], [15].

### Study population

Patients with an initial documentation of a workplace conflict experience (coded as “Discord with boss and workmates” (Z56.4)) between January 2005 and December 2014 were identified in 699 general practitioner practices (index date). From 12,853 patients with ICD Code Z56.4, in 7,374 patients, we found a physician’s original note containing the term ‘mobbing’ additionally to ICD Code Z56.4. We included only patients who had both documentation of ICD Code Z56.4 and a physician’s original note containing the term ‘mobbing’.

These physician notes were essential since the ICD-10 code used comprises different problems and not just workplace conflicts.

**Figure 1:**
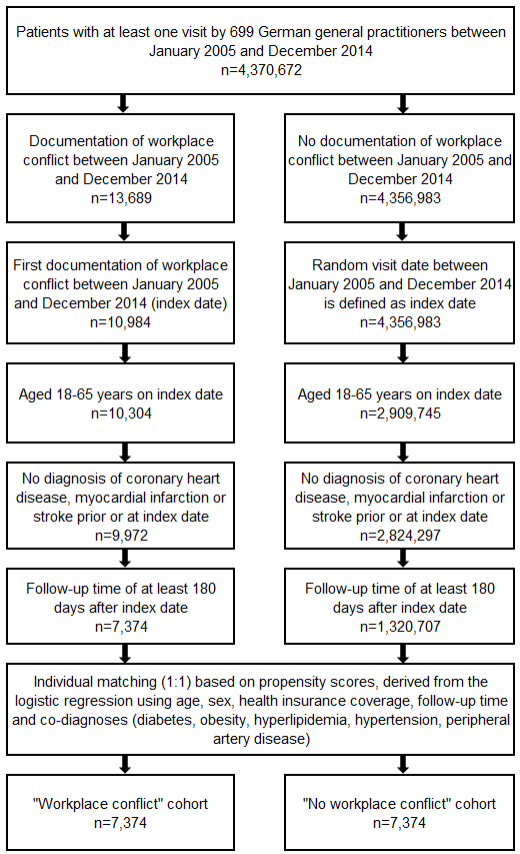
Selection of study patients

We included only those that were between the ages of 18 and 65 years, had a follow-up time of at least 180 days after the index date, and had not been diagnosed with angina pectoris, myocardial infarction, coronary heart diseases (ICD-10: I20-24), or stroke (ICD-10: I63, I64, G45) prior to the documentation of the workplace conflict. In total, 7,374 patients in the ‘workplace conflict’ cohort were available for analysis (Figure 1 (Fig. 1)). Each patient that experienced conflict was matched (1:1) to a control without conflict experience based on propensity scores derived from the logistic regression using physician, age, sex, health insurance coverage, follow-up duration, and co-diagnoses (diabetes (ICD-10: E10-14), obesity (E66), hyperlipidemia (E78), hypertension (I10), and peripheral artery disease (E70, E73.9)). We also found 7,374 patients in the control group for analysis.

### Study outcome

The main outcome measure was the incidence of angina pectoris (I20), myocardial infarction (I21-23, I25.2), and stroke (I63, I64, G45) correlated with workplace conflict experiences in German general practitioner practices.

### Statistical analyses

Descriptive analyses were obtained for age, sex, health insurance coverage, follow-up duration, and co-diagnoses (diabetes, obesity, hyperlipidemia, hypertension, and peripheral artery disease). Kaplan-Meier curves were used to analyze the proportion of patients with cardiovascular diseases over time in each group (patients with and without conflict experience). Finally, a multivariable Cox regression model was used to analyze the association between cardiovascular diseases and workplace conflict experience. P-values <0.05 were considered statistically significant. Analyses were carried out using SAS version 9.4. 

## Results

**Figure 2:**
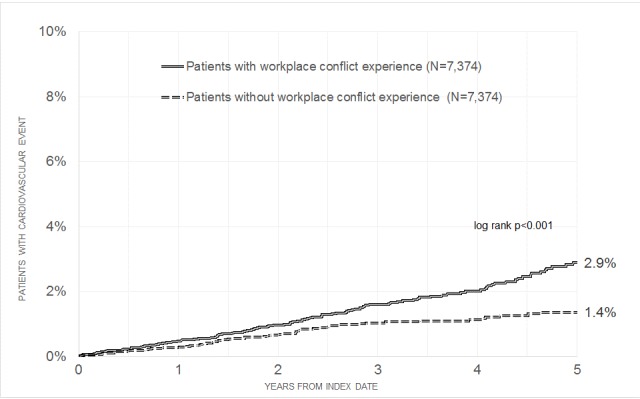
Kaplan-Meier curves for time to the diagnosis of cardiovascular disorders in primary care patients with workplace conflict experience and matched controls

The baseline characteristics of patients included in the present study are displayed in Table 1 (Tab. 1). After individual matching, mean age was 37.5 years (SD=12.4 years), 33.0% were men, and 3.0% had private health insurance coverage. The mean follow-up lasted 3.4 years (SD=2.2 years). The most frequent disorder at baseline was hypertension (13.7%). Figure 2 (Fig. 2) shows Kaplan-Meier curves for the time to the diagnosis of cardiovascular diseases in patients with a workplace conflict experience and in controls. After a maximum of five years of follow-up, 2.9% of the individuals with workplace conflict experience had been affected by cardiovascular diseases compared with 1.4% in the control group (p-value <0.001). The results of the Cox regression model analyses are displayed in Table 2 (Tab. 2). Workplace conflict was associated with a 1.63-fold increased risk of developing cardiovascular diseases (95% CI: 1.24–2.15). Risk of cardiovascular events was higher in men and obese people. Finally, the impact of workplace conflict was significant for angina pectoris (OR=1.79, 95% CI: 1.22–2.64), but not for myocardial infarction (OR=2.03, 95% CI: 0.91–4.51) or stroke (OR=1.56, 95% CI: 0.96–2.56). 

## Discussion

 This analysis of routine healthcare data, including 7,374 cases and 7,374 controls, showed that workplace conflict experiences were associated with an increased risk of developing cardiovascular disorders. The impact of workplace conflicts was significant on angina pectoris, but not on myocardial infarction or stroke. Cardiovascular diseases were also significantly associated with age, sex, and obesity. 

Workplace conflicts including mobbing are known to have a negative impact on health and are well known to increase the risk of depression, anxiety and sleep disorders [6], [7], [8]. By contrast, few studies have focused on the association between workplace mobbing and negative cardiovascular outcomes. In 2003, Kivimäki et al. showed in a cohort of 5,432 hospital employees from Finland that 2% of the population reported bullying experiences [9]. Workplace mobbing was associated with a 2.3-fold increased risk of being subsequently diagnosed with cardiovascular diseases. Interestingly, the same study found that prolonged bullying also had an impact on depression. Later, in 2005, Malinauskienë et al. investigated the prevalence of workplace psychological terror in Lithuanian secondary school teachers and the effect of bullying on stress and negative cardiovascular outcomes [10]. They discovered that the prevalence of regular bullying was 2.6% and that of occasional bullying was 23.0%. After adjusting for age and gender, workplace mobbing was positively associated with stress symptoms (OR=2.97). Finally, the odds ratio of mobbing for cardiovascular diseases was 1.31 (95% CI: 1.01–1.72), although it became non-significant after adjusting the regression model for age, gender, smoking, and being overweight (OR=1.32, 95% CI: 0.99–1.77). More recently, in 2010, Tuckey et al. examined the risk of poor mental and cardiovascular health associated with past and current exposure to workplace mobbing in 251 police officers from Australian police [11]. The authors found significant correlations between past exposure to bullying and two indicators of poor cardiovascular health (high blood pressure and frequent cardiac consultation), as well as between current exposure and poor mental health. 

Depression and anxiety may play an important role in the association between workplace conflicts and cardiovascular disorders. People who are victims of bullying at work are more likely to be depressed and anxious and are thus indirectly more likely to develop cardiovascular diseases. In 2014, Kostev et al. discovered that depression, anxiety, somatoform disorders, and sleep disorders were more frequent in individuals with workplace mobbing experiences than in controls free of this negative experience [7]. That same year, a European study found that self-reported symptoms of depression and anxiety, especially if recurrent, are associated with an increase in the risk of developing acute myocardial infarction [16]. These results corroborated previous works that had found that depression was a risk factor for the onset of a wide range of cardiovascular disorders [17], [18], [19]. The relationship between depression/anxiety and cardiovascular diseases involves an alteration of numerous biological systems, pathways, and molecules, such as the autonomic nervous system, platelet receptors, coagulopathic factors (i.e. plasminogen activator inhibitor-1 or fibrinogen), pro-inflammatory cytokins, endothelial function, and neurohormonal factors [20]. Taking all these considerations into account, one way to reduce the risk of negative cardiovascular outcomes in ‘victims’ of workplace conflicts would be to prescribe medications for depression. In line with this hypothesis and despite some concerns about the safety of the long-term use of antidepressants, it has been discovered that depressed individuals receiving antidepressants for 12 weeks or more have a lower risk of myocardial infarction than depressed individuals with no antidepressant therapy [21].

Another important result of the present retrospective German study is that the impact of workplace conflicts was significant on angina pectoris but not on myocardial infarction or stroke. As myocardial infarction and stroke are less frequent than angina pectoris, the differences in the significance can result from the lower statistical power of myocardial infarction and stroke compared to that of angina pectoris in this study. 

Since these findings are new, they must be interpreted with considerable caution. Nonetheless, they can highlight the fact that the effect of workplace conflicts on cardiovascular disorders is not homogenous but varies from one disease to another. Interestingly, such difference in the risk of being diagnosed with one particular cardiovascular disorder has also been found in two recent European studies, as the impact of depression on several cardiovascular conditions was not identical [22], [23]. Finally, age, sex, and obesity were also significantly associated with these diseases. These last findings are in line with the present literature, as older people, men, and obese individuals are more likely to be diagnosed with heart diseases than younger people, women, and non-obese individuals [24], [25], [26].

Retrospective primary care database analyses are generally limited by the validity and completeness of the data on which they are based. The present study included several limitations, such as the assessment of workplace conflict experience, since this documentation may not be complete. Workplace conflict is not a diagnosis, and a doctor will only know about it when patients report it to them. Furthermore, data pertaining to socioeconomic status (e.g., education and income) and lifestyle-related risk factors (e.g., smoking, alcohol, and physical activity) were lacking. Moreover, information about patients’ employment status was missing. Even if the age of patients with and without workplace conflict experience is the same, it is possible that the share of unemployed patients in the control group is higher, which could impact our results.

Furthermore, the data only included patients treated by general practitioners, and no information from other physicians or hospitals was available. Finally, the database does not include information about mortality. Therefore, only non-fatal cardiovascular events could be included in the study. On the other hand, the study had several strengths. More than 14,500 German individuals were available for analysis and several comorbidities were included in the regression model. Moreover, the database used provides information on various professions, even if these professions are not listed individually. Second, the information on cardiovascular outcomes is not based on self-report but on diagnoses from general practitioners. Furthermore, separate odds ratios for stroke, myocardial infarction, and angina pectoris, not just for cardiovascular outcomes as a whole, are provided.

Overall, the present study showed that workplace conflict experience was associated with cardiovascular disorders. However, further research is needed to gain a better understanding of the impact of workplace situation on these negative cardiovascular outcomes. 

## Data

Data for this article are available from the Dryad Repository: http://dx.doi.org/10.5061/dryad.178vs [27].

## Notes

### Competing interests

Karel Kostev is an employee of IMS Health. IMS Health (http://www.imshealth.de/sites/en/about-us/our-company) is a commercial research institute providing information, services, and technology for the healthcare industry. The authors declare that they have no competing interests. 

### Ethical approval

This article does not contain any studies with human participants performed by any of the authors.

## Figures and Tables

**Table 1:**
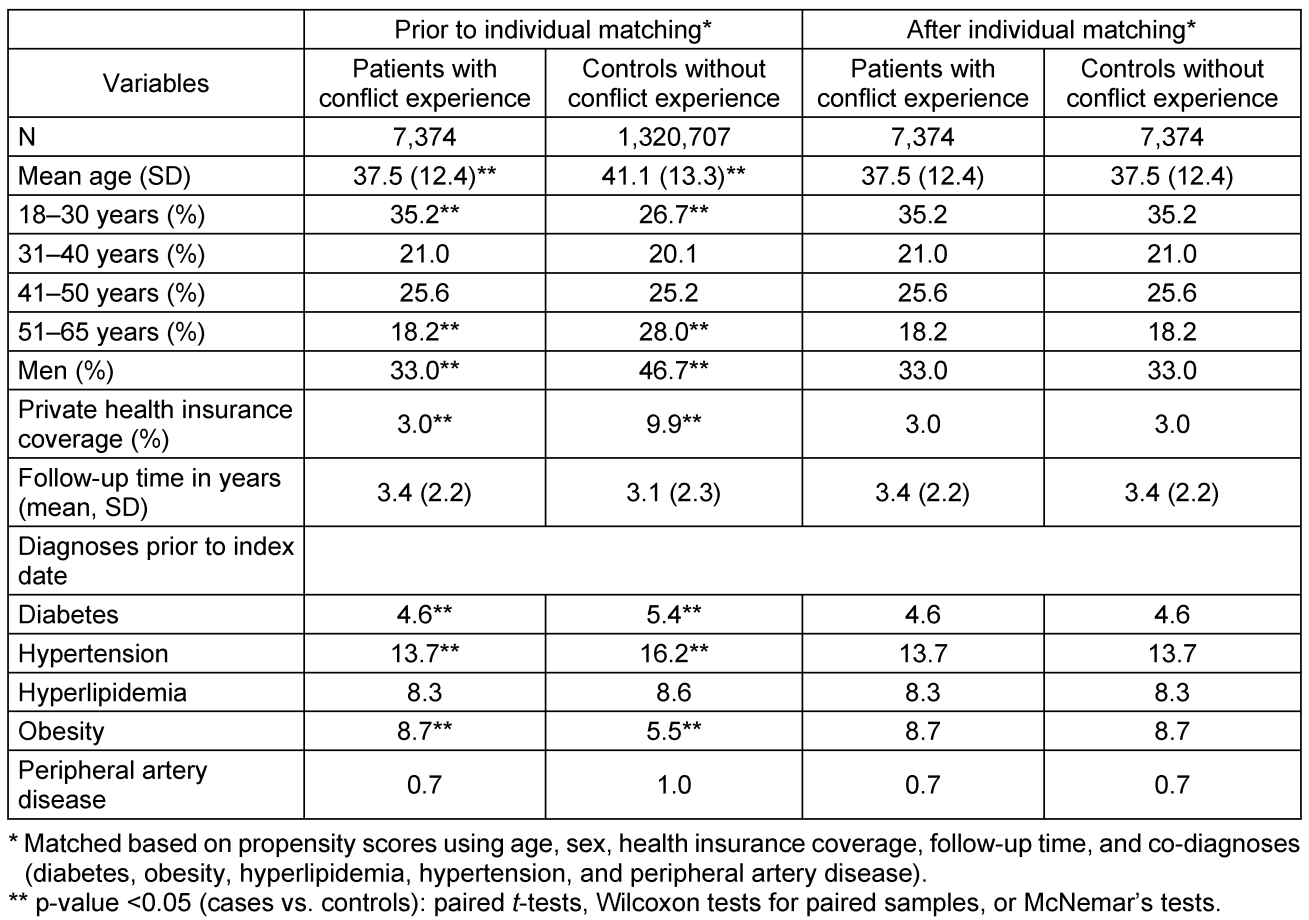
Baseline characteristics of patients with and without workplace conflict experience treated in German GP practices prior to and after individual matching

**Table 2:**
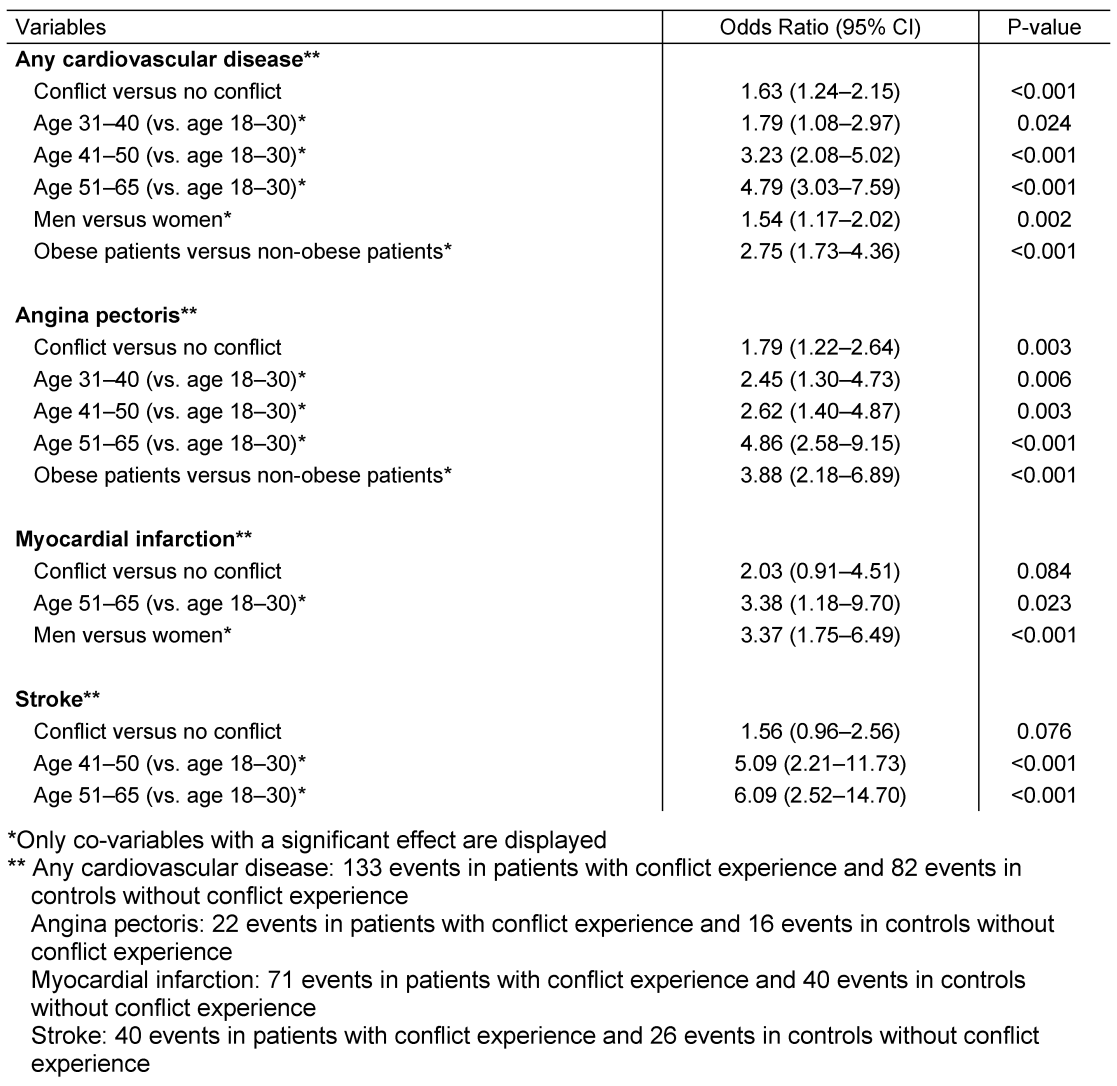
Association between workplace conflict experience and cardiovascular disorders
